# Cerebral White Matter Mediation of Age-Related Differences in Picture Naming Across Adulthood

**DOI:** 10.1162/nol_a_00065

**Published:** 2022-03-30

**Authors:** Sara B. W. Troutman, David J. Madden, Michele T. Diaz

**Affiliations:** Department of Psychology, Pennsylvania State University, University Park, PA, USA; Brain Imaging and Analysis Center & Department of Psychiatry and Behavioral Sciences, Duke University Medical Center, Durham, NC, USA; Center for Cognitive Neuroscience, Duke University, Durham, NC, USA; Social, Life, & Engineering Sciences Imaging Center, Pennsylvania State University, University Park, PA, USA

**Keywords:** diffusion tensor imaging, picture naming, aging, language production

## Abstract

As people age, one of the most common complaints is difficulty with word retrieval. A wealth of behavioral research confirms such age-related language production deficits, yet the structural neural differences that relate to age-related language production deficits remains an open area of exploration. Therefore, the present study used a large sample of healthy adults across adulthood to investigate how age-related white matter differences in three key left-hemisphere language tracts may contribute to age-related differences in language ability. Specifically, we used diffusion tensor imaging to measure fractional anisotropy (FA) and radial diffusivity (RD) which are indicators of white matter structure. We then used a series of path models to test whether white matter from the superior longitudinal fasciculus (SLF), the inferior longitudinal fasciculus, and the frontal aslant tract (FAT) mediated age-related differences in one form of language production, picture naming. We found that FA, as well as RD from the SLF and FAT mediated the relation between age and picture naming performance, whereas a control tract (corticospinal) was not a mediator. Moreover, differences between mediation of picture naming and a control naming condition suggest that left SLF has a greater role in higher-order aspects of naming, such as semantic and lexical selection whereas left FAT is more sensitive to sensorimotor aspects of fluency or speech motor planning. These results suggest that dorsal white matter contributes to age-related differences in generating speech and may be particularly important in supporting word retrieval across adulthood.

## INTRODUCTION

One of the most common age-related complaints is word retrieval difficulty (Ossher et al., 2013). Such age-related challenges in language production are well documented (Burke & Shafto, 2008): Compared to younger cohorts, older adults produce speech more slowly (Gollan et al., 2008), make more errors (Feyereisen, 1997), use more filler words and pauses (Kemper et al., 1992), produce more off-topic speech (Arbuckle & Gold, 1993), and experience more tip-of-the-tongue events and word finding difficulties (Burke et al., 1991). Behavioral research suggests that age-related deficits in language production may be related to deficits in accessing the sounds of words. In the context of the transmission deficit hypothesis (Burke et al., 1991), age-related weakening among phonological connections may underlie language production deficits. Although recent functional magnetic resonance imaging (MRI) research found similar neural and behavioral sensitivity to phonological characteristics for younger and older adults, age-related differences in picture naming across adulthood were associated with increases in functional activation (Diaz et al., 2021). However, it remains unclear how brain structure may relate to these age-related language production deficits.

One possible neural mechanism for age-related increases in language production difficulty is the structural degradation of cerebral white matter, which comprises the myelin-coated axonal fibers conveying signals across the brain. Information regarding cerebral white matter can be obtained by MRI through susceptibility-weighted data. Diffusion tensor imaging (DTI) provides information regarding the rate and directionality of molecular water motion at the voxel level (Basser, 1995; Jones et al., 2013; Pierpaoli et al., 1996; A. W. Song et al., 1996; S.-K. Song et al., 2003, 2005). Within a tensor model, for example, fractional anisotropy (FA) reflects the degree to which diffusivity is directional rather than random (isotropic), and radial diffusivity (RD) reflects the rate of diffusivity perpendicular to the principal direction (eigenvalue) of the tensor. Thus, assuming that many white matter fibers contribute to the estimates obtained from each voxel, higher spatial coherence of fibers would lead to increasing FA (i.e., higher directionality of diffusion), and a greater hindrance to diffusion across the fibers, provided by more complete myelination, would lead to lower RD (S.-K. Song et al., 2003, 2005). Thus, these DTI measures are informative regarding the underlying structural integrity of white matter, despite the fact that these measures are indirect and influenced by other variables (Jones et al., 2013; Wheeler-Kingshott & Cercignani, 2009).

Structural disconnection, specifically changes in the white matter tracts that emerge through aging, have been linked to age-related differences in several areas of cognition, especially in measures of executive function and processing speed (Bennett & Madden, 2014; Gazes et al., 2016; Hedden et al., 2016; Johnson et al., 2015; Salami et al., 2012), though the degree to which white matter has an independent or interactive effect with age is unclear (Madden et al., 2017, 2020; Salami et al., 2012). Moreover, a number of studies have pointed to the importance of white matter connections in language production ability (De Zubicaray et al., 2011; Dick et al., 2014; Houston et al., 2019; Madhavan et al., 2014; Stamatakis et al., 2011; Troutman & Diaz, 2019). Studies using DTI-derived metrics of white matter structure have shown that white matter along several key tracts within the language network are sensitive to age-related differences. These tracts include the superior longitudinal fasciculus-III (SLF-III; Houston et al., 2019; Madhavan et al., 2014; Stamatakis et al., 2011; Troutman & Diaz, 2019), the inferior longitudinal fasciculus (ILF; Kantarci et al., 2011; Stamatakis et al., 2011; Troutman & Diaz, 2019), and the frontal aslant tract (FAT; Catani et al., 2013; Rizio & Diaz, 2016; Troutman & Diaz, 2019). It is important to note that there are several models of these tracts (for reviews, see Dick et al., 2014; Dick & Tremblay, 2012; Friederici, 2009, 2012; Varriano et al., 2020). For example, the SLF-III is in close proximity to the arcuate fasciculus, which also supports language (e.g., Perron et al., 2021; Tremblay et al., 2019), and there is debate about where each terminates posteriorly (e.g., posterior parietal, posterior superior temporal gyrus). With respect to the FAT, which connects inferior frontal gyrus regions with supplemental motor regions (SMA & pre-SMA) in superior frontal gyrus, it has recently been suggested that there is an additional anterior portion that may play a role in working memory (Varriano et al., 2020).

Several groups have reported that, among both younger and older adults, higher FA along the left SLF correlated with better naming performance (Houston et al., 2019; Madhavan et al., 2014; Stamatakis et al., 2011; Troutman & Diaz, 2019; for a review see Dick et al., 2014; Dick & Tremblay, 2012). Moreover, Stamatakis et al. (2011) found that age-related word-finding failures were linked to lower FA, particularly in the posterior portion of the left SLF. This suggests that white matter deficits, particularly along the left posterior SLF, may play a role in driving age-related language production deficits. Madhavan et al. (2014) and Houston et al. (2019) found that lower FA across the SLF was linked to poorer performance on clinical tests of language production (i.e., the Controlled Oral Word Association Test (Benton et al., 1983) and the Boston Naming Test (Kaplan et al., 1983), respectively). More recently, Troutman and Diaz (2019) studied white matter using both FA and RD. They found that higher FA within dorsal tracts, including the SLF and FAT, was linked to better picture naming during a phonological picture-word interference task. While the effect of FA in their study was localized to dorsal tracts, specifically the SLF and FAT, the effect of RD was more widespread. Language production performance was linked to RD along dorsal language tracts (the SLF and FAT), as well as ventral language tracts (the ILF and middle longitudinal fasciculus) and the frontostriatal tract. That is, participants with higher FA along dorsal language tracts and lower RD along all tracts named pictures the most accurately. Importantly, effects of age and white matter shared variance in explaining behavioral differences in picture naming, suggesting that age-related declines in white matter, particularly from dorsal pathways, have a substantive role in age-related language production deficits.

### The Current Study

We sought to extend prior studies examining the relations between aging, language production, and white matter in several ways. First, we took a more mechanistic approach by investigating the influence of white matter on the relation between age and language production with mediation analyses by building a series of path models to test the role of the SLF, FAT, ILF, and a control tract, the corticospinal (CS) tract on naming performance. Second, we examined the role of white matter and age by comparing naming under two conditions (object naming and repeating a word to an abstract image). This allowed us to distinguish between sensorimotor aspects of naming (e.g., motor control, articulation) from lexical and semantic aspects of naming. Third, we included a broad sample of adults of different ages, who completed a picture-naming task and MRI scanning, including diffusion-weighted imaging (DWI), to allow us to examine these relations across adulthood.

Given the prior literature, we hypothesized that dorsal language tracts (SLF, FAT) would be most important for selection and retrieval aspects of picture naming, and that FA would discriminate naming performance between picture naming and our control condition. Because previous research has found effects of RD on picture naming within both dorsal and ventral language tracts, we hypothesized that RD might be sensitive to naming in general (i.e., across conditions). We expected higher FA (i.e., more cohesive white matter) and lower RD (i.e., more myelinated tracts) would predict faster and more accurate picture naming. We also expected that age would be negatively correlated with FA and positively correlated with RD, reflecting well-documented age-related deficits in white matter. Critically, we hypothesized a mediating path, whereby the effect of dorsal white matter would mediate the effect of age on picture naming, such that increases in FA and decreases in RD would be associated with better picture naming performance.

## MATERIALS AND METHODS

### Participants

Ninety-three healthy, right-handed, monolingual, native American-English speaking adults participated in this study. Participants were community-dwelling adults, recruited from campus and the local area via radio ads and flyers. Data from one participant were removed because of poor picture naming performance (>3 *SD*s from the mean) and a second participant’s data were removed due to a high score on our depression screening (Geriatric Depression Scale; Guerin et al., 2018; Sheikh & Yesavage, 1986). The final sample included data from 91 participants aged 20–75 (mean age = 47.40 years, *SD* = 17.45 years), 54 female, 37 male. All participants had normal or corrected-to-normal vision as indicated by the Freiburg Visual Acuity Test (Bach, 1996). Participants reported no history of neurological, psychological, or major medical conditions nor did they report any contraindications to MRI scanning (e.g., having a pacemaker; Christensen et al., 1992). Participants also completed a battery of psychometric and neuropsychological tests to assess cognitive functions such as speed, working memory, executive function, and language. Participant demographics and cognitive scores are reported in Table 1. A detailed description of the battery is available in Diaz et al. (2021), where results from the fMRI analysis are also reported. All procedures were approved by the Pennsylvania State University Institutional Review Board, and all participants provided written, informed consent.

**Table 1.** Participant demographic and neuropsychological testing scores

Demographic information	
*N*	91
Age (years)	47.40 (20–75, 17.45)
Gender (M/F)	37/54
Education (years)	16.9 (12–25, 2.5)

Cognitive assessments–age correlation	
Education	0.24*
MMSE	−0.19
Depression (GDS)	−0.13
Speed RT (choice)	0.56***
WAIS-III digit symbol RT	0.69***
WAIS-III digit span forward	−0.20
WAIS-III digit span backward	−0.26*
Stroop effect	0.33***
Verbal working memory	−0.40***
CVLT immediate recall	−0.27*
CVLT delayed recall	−0.26*
Category fluency (animals)	−0.30**
Phonemic fluency (F, A, S)	−0.11
WAIS-III vocabulary	0.07
Author recognition task	0.47***

*Note*. The color Stroop task was used; the Stroop effect was the difference in reaction times (RT) between word-font incongruent minus word-font congruent trials. The author recognition task (Acheson et al., 2008) uses author name recognition to assess reading habits, which may be less biased compared to self-report. Demographic information for age and education is the mean, with range and standard deviation in parentheses. MMSE = Mini-Mental State Exam (Folstein et al., 1975); GDS = Geriatric Depression Scale (Guerin et al., 2018; Sheikh & Yesavage, 1986); WAIS-III = Wechsler Adult Intelligence Scale (Wechsler, 1997); CVLT = California Verbal Learning Test (Delis et al., 1987). *p < 0.05, **p < 0.01, ***p < 0.001.

### Stimuli and Procedure

During their functional MRI scan, participants named a series of pictures and said “picture” to abstract images (191 color photographs of objects and 50 diffeomorphically scrambled control images (Stojanoski & Cusack, 2014), 396 pixels × 396 pixels, duration = 1,500 ms). Photographs were of everyday objects (e.g., animals, fruit, vehicles, household objects, etc.), and were largely selected from normed databases (Brodeur et al., 2010, 2014; Moreno-Martinez & Montoro, 2012). All photographs underwent a separate norming procedure with a different group of younger participants (*N* = 28; mean age = 19.48, *SD* = 1.37; 16 females) to ensure high name agreement (mean H-index = 0.25, *SD* = 0.40, range = 0–1.55; H-index is a measure of name agreement that accounts for the variability in acceptable names given by participants for a particular image by considering both the number of acceptable names and their proportions [Snodgrass & Vanderwart, 1980]; lower H-index values correspond to higher name agreement). Photographs were presented in a random order with a variable inter-stimulus interval (range = 1.5–15 s, mean = 3.40 s) to optimize the hemodynamic response (Dale, 1999). Responses were recorded and filtered using an MR-compatible, dual-channel, fiber-optic microphone system (Optoacoustics Ltd., Or-Yehuda, Israel). For additional details on the stimuli and task procedures see Diaz et al. (2021).

### Behavioral Data Analyses

Responses to the photographs of objects were considered to be correct if the participant provided the anticipated picture name (e.g., *airplane* for *airplane*), the plural form of an otherwise correct name, or an acceptable alternative (e.g., *jet* for *airplane*). Across all participants, this method yielded a total of 17,381 observations before data cleaning, including 12,698 modal answers, 1,629 acceptable alternatives, and 3,054 incorrect answers. In cases where participants provided an acceptable alternative, word characteristics such as length and frequency were calculated for the provided response. Alternate names were, on average, more frequent, shorter, and had more phonological neighbors.

For naming latencies, only correct trials as defined above were considered. To calculate naming latencies we used custom scripts in PRAAT (Boersma, 2001). These scripts used pitch and intensity cues to identify potential word onsets, which were manually verified by trained research assistants (additional details can be found in the Supporting Information at https://doi.org/10.1162/nol_a_00065). Then, we calculated naming latencies by taking the difference between the stimulus onset and the onset of the participant’s response. We excluded trials where the naming latency was implausibly low or not recorded (i.e., >200 ms; *n* = 219) or if the naming latency constituted an outlier (i.e., greater than or less than 2.5 *SD*s from that participant’s mean naming latency or mean accuracy; *n* = 636). Thus, there were a total of 855 excluded outliers (∼4.92% of total responses), leaving a final data set of 13,472 total picture naming observations.

In order to minimize the influence of speed-accuracy trade-offs in the analysis, we calculated an inverse efficiency score (IES) at the participant level (Horowitz & Wolfe, 2003). We constructed the IESs by dividing each participant’s average naming latency by their average accuracy. Similar to naming latencies, a higher IES indicates slower naming while a lower IES indicates faster naming, but an IES adjusts latency by the associated accuracy. Responses to control images were considered correct if participants replied “picture” to the scrambled image as per the instructions. In total, participants accurately identified 4,437 scrambled images. Trials with implausible or no response times (i.e., <200 ms, *n* = 138) and subject-level outliers (*n* = 230) were removed, leaving a total of 4,182 control trials. Analysis scripts along with behavioral and DTI data are available via the Open Science Framework: https://osf.io/3pxkw/.

### Acquisition of MRI Data

A 3T Siemens Prisma Fit MRI scanner and a 64-channel head coil were used to collect the MRI data. Sagittal T1-weighted localizer images were used to identify a volume for data collection, shimming, and alignment to the anterior and posterior commissures (AC-PC). A magnetization-prepared rapid acquisition gradient echo (MPRAGE) sequence was used to collect T1-weighted anatomical images (repetition time [TR] = 2,300 ms; echo time [TE] = 2.28 ms; inversion time [TI] = 900 ms; flip angle = 8°; echo spacing = 7 ms; acceleration factor = 2; field of view [FOV] = 256 mm^2^; voxel size = 1 mm^3^; 160 contiguous slices; duration ∼5 min).

Diffusion weighted data were acquired using 64 diffusion-weighted directions (b-value = 1,000 s/mm^2^, TR = 5,520 ms; TE = 89.40 ms; echo spacing = 0.62 ms; flip angle = 78°; FOV = 240 mm^2^; voxel size = 2 mm^3^; 72 contiguous slices; acceleration factor = 2; fat saturation was used; anterior to posterior phase encoding). Three non-diffusion-weighted b0 acquisitions (0 s/mm^2^) were collected at the beginning, middle, and end of the sequence. Immediately following the main diffusion sequence, one diffusion weighted volume was also collected with the same parameters using a posterior to anterior phase encoding direction to correct for field inhomogeneities.

### DTI Preprocessing and Tractography

Data were preprocessed using MRTRIX (Tournier et al., 2019) and FSL Tools (Smith et al., 2004) and visually inspected for quality. During preprocessing, data were first denoised using the MRTRIX command dwidenoise, which uses principal component analysis to identify noise-only components (Veraart et al., 2016). Second, to correct for artifacts such as eddy currents and susceptibility artifacts, data were preprocessed using the MRTRIX command dwifslpreproc, which incorporates FSL’s Topup and eddy tools (Andersson et al., 2003). Next, data were skull-stripped using Brain Extraction Tool (Smith, 2002). Finally, to correct for encoding bias, data were field corrected using the MRTRIX command dwibiascorrect. Voxel-wise tensors and tensor metrics were estimated using the MRTRIX commands dwi2tensor and tensor2metric, respectively. Data were linearly registered to the MNI152 2mm template using FSL FLIRT (Jenkinson et al., 2002). Three left-hemisphere tracts of interest, the SLF, FAT, and ILF tracts, as well as one control tract, the CS tract, were identified using probabilistic tractography (Smith et al., 2004; Tournier et al., 2019). All tractography was run in each individual’s native space. Seeds and targets were warped into native space using FSL (Supplemental Table 1; Greve & Fischl, 2009). Seeds and target are detailed in Supplemental Table 1 and tracts were estimated using the MRTRIX command tckgen using the Tensor_prob algorithm with the default parameters (i.e., 5,000 streamlines, tract length 5–100 voxels).

**Figure 1.**
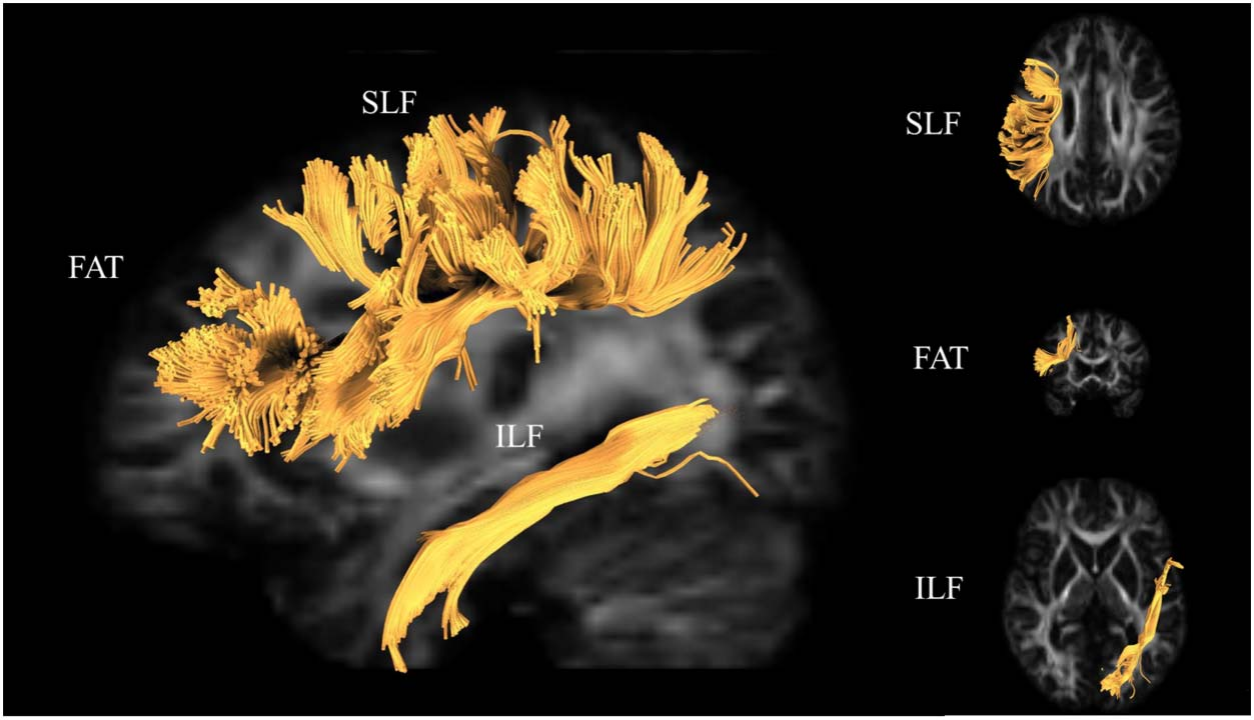
Representative illustrations of the tracts of interest. The superior longitudinal fasciculus (SLF), the inferior longitudinal fasciculus (ILF), and the frontal aslant tract (FAT) are depicted above. These tracts were chosen because they represent tracts that have known associations with language production ability. One additional tract, the corticospinal tract (CS; not pictured) was also included as a control tract to test whether the results were specific to the language tracts.

At each voxel, the Tensor_prob algorithm bootstraps DWI data via trilinear interpolation, and the resultant principal eigenvector is used to guide the streamline at that step. A valid streamline is one that begins at a randomly selected voxel from the seed region and continues until reaching a voxel in the target region without exiting the brain or crossing into the opposite hemisphere. Representative examples of each tract are displayed in Figure 1. To minimize directional bias resulting from starting the tracking procedure in a given seed, the tracking algorithm was run twice for each tract: once beginning in the seed and ending in the target and once beginning in the target and ending in the seed. These tracts were then combined, keeping only voxels common to both tracking directions. Tracts were manually inspected for quality such that each viable tract passed through at least three contiguous slices in the most optimal plane of view for the given tract. Because probabilistic tractography can result in a small number of spurious tracts, tracts were cleaned based on a priori anatomical knowledge (Catani & de Schotten, 2012) to remove extraneously included paths based on the criteria in Supplemental Table 1. Finally, average FA and RD values were estimated for each participant’s SLF, ILF, FAT, and CS. We selected tracts from the left hemisphere because language production tends to be left lateralized in right-handed individuals.

### Path Model Testing

**Figure 2.**
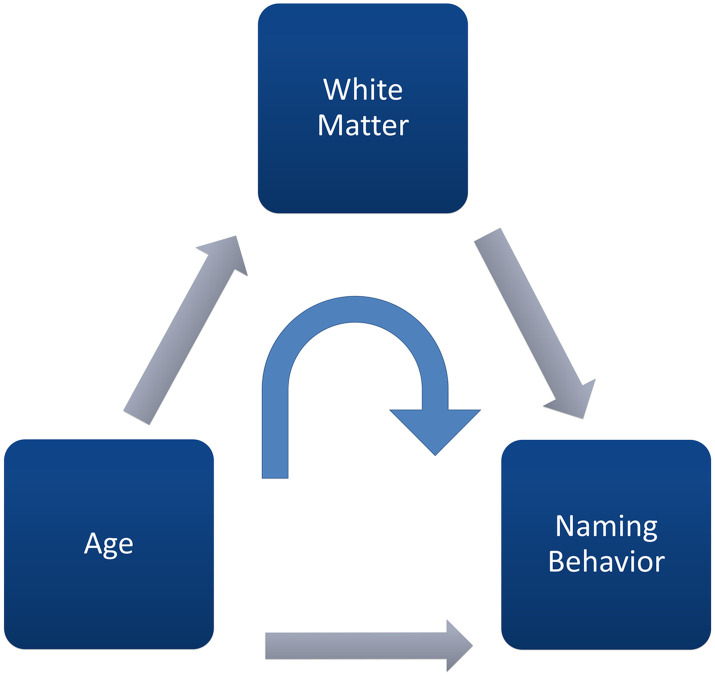
Representative illustration of the path models used in this analysis to examine the relations between age, white matter, and inverse efficiency scores (IESs). Grey arrows depict main effects and blue arrows depict mediating paths.

Path analyses were run in RStudio (R Core Team, 2014; RStudio Team, 2020) using the lavaan package (Rosseel, 2012). Two separate models (i.e., one for FA and one for RD) were created for each of the four tracts (i.e., the SLF, ILF, FAT, and CS), resulting in a total of eight path models. All eight models follow the same basic model depicted in Figure 2. All the models include the same terms: the main effects of white matter (i.e., FA or RD from a given tract); the main effect of age on picture naming latency (IES scores); the effect of age on white matter; and white matter mediating the relationship between age and picture naming. Model fit metrics indicated an acceptable model fit in all cases (comparative fit index [CFI] >0.9; Tucker–Lewis index [TLI] >0.90; root mean square error of approximation [RMSEA] <0.08; see Supplemental Table 2 for details; Hooper et al., 2008). To minimize the influence of false positives, we used a Bonferroni-corrected significance threshold of *p* = 0.006 (i.e., 0.05 / 8 = 0.006). To compare the strength of relationships between paths, we used Fisher *r* to *z* transformations.

## RESULTS

### Behavioral Results

Consistent with age-related slowing, IESs were positively correlated with age (*t* = 36.74, *p* < 0.0001). There was no significant main effect of age on naming time. However, age was associated with lower accuracies (*t* = −2.92, *p* < 0.005, eta squared = −0.08), and there was a marginally significant quadratic effect of age on accuracy (*p* = 0.08), with the strongest effect of age on accuracy among the oldest adults. Thus, the age-related differences in IESs were most likely driven by age-related differences in accuracy.

### Fractional Anisotropy

Consistent with age-related slowing, IESs were positively correlated with age (*t* = 36.74, *p* < 0.0001); for additional details see Supporting Information. With respect to the DTI measures, simple bivariate correlations confirmed that increases in FA were related to faster naming. In the path models, age was negatively related to FA from all tracts: the SLF (β = −0.092, *SE* = 0.009, *p* < 0.001), ILF (β = −0.131, *SE* = 0.009, *p* < 0.001), FAT (β = −0.073, *SE* = 0.01, *p* < 0.001), and CS (β = −0.030, *SE* = 0.01, *p* = 0.002). Picture naming latency increased with age in most models: SLF (β = 62.89, *SE* = 1.56, *p* < 0.001), ILF (β = 31.95, *SE* = 1.73, *p* < 0.001), FAT (β = −0.073, *SE* = 0.009, *p* < 0.001), and CS (β = 56.33, *SE* = 1.65, *p* < 0.001). See Table 2 for model details.

**Table 2.** Path model estimates for the relationship between age, FA, and naming

Naming pictures of everyday objects				
	SLF	ILF	FAT	CS
Age–FA	−0.092 (0.009)*	−0.131 (0.009)*	−0.073 (0.01)*	−0.030 (0.01)*
Age–IES	62.89 (1.56)*	31.95 (1.73)*	−0.073 (0.01)*	56.33 (1.65)*
FA–IES	13.55 (1.51)*	−0.004 (1.53)	−9.64 (1.63)*	−7.16 (1.62)*
FA mediation of Age–IES	−1.26 (0.19)*	0.099 (0.20)	0.71 (0.15)*	−0.93 (0.46)
Naming abstract control items				
	SLF	ILF	FAT	CS
FA–IES	−2.76 (1.66)	0.29 (1.76)	24.78 (1.30)*	−7.78 (1.80)*
FA mediation of Age–IES	0.25 (0.16)	−0.04 (0.25)	−2.02 (0.44)*	0.23 (0.15)

*Note*. Values provided are beta estimates with standard errors in parentheses. *p < 0.006, for statistically significant relationships, the significance threshold accounts for multiple comparisons.

FA from the SLF, FAT, and CS were related to picture naming latency (β = 13.55, *SE* = 1.51, *p* < 0.001; β = −9.64, *SE* = 1.63, *p* < 0.001; β = −7.16, *SE* = 1.62, *p* < 0.001). Moreover, FA from the SLF and the FAT mediated the relation between age and naming latency (β = −1.26, *SE* = 0.19, *p* < 0.001; β = 0.71, *SE* = 0.15, *p* < 0.001). FA from the CS did not (*p* = 0.01). Importantly, the relationships between picture naming and SLF FA and picture naming and FAT FA were significantly stronger than the corresponding effect of FA from the CS control tract (*z* = 7.092, *p* < 0.001; *z* = 2.810, *p* = 0.005). There were no significant relationships between picture naming and FA from the ILF (β = −0.004, *SE* = 1.53, *p* = 0.613), nor did this mediate the relationship between age and picture naming.

The role of FA on the control condition (i.e., saying “picture” in response to abstract images) revealed that FAT FA was positively related to naming the control trials (β = 24.78, *SE* = 1.30, *p* < 0.001) and FAT FA mediated the relationship between age and naming control trials (β = −2.02, *SE* = 0.44, *p* < 0.001). Though, CS FA was related to naming latency (β = −7.78, *SE* = 1.80, *p* < 0.001), the relationship between age and naming control trials was not mediated by FA from the CS (*p* = 0.124). Moreover, the mediating effect of FAT FA was significantly greater than the corresponding relationship between CS FA and naming latency (*z* = 4.875, *p* < 0.001). FA from the SLF and ILF did not show significant relationships with the control condition naming latency (*p*s > 0.10), nor were these significant mediators (*p*s > 0.09).

### Radial Diffusivity

Simple bivariate correlations indicated that increases in RD were associated with slower naming times. In the path models, age was positively related to RD from all models: the SLF (β = 0.37, *SE* = 0.009, *p* < 0.001), ILF (β = 0.08, *SE* = 0.009, *p* < 0.001), FAT (β = 0.42, *SE* = 0.009, *p* < 0.001), and CS (β = 0.28, *SE* = 0.01, *p* < 0.001). See Table 3 for model details. Picture naming latency increased with age in all models: SLF (β = 66.96, *SE* = 1.70, *p* < 0.001), ILF (β = 52.42, *SE* = 1.52, *p* < 0.001), FAT (β = 60.09, *SE* = 1.70, *p* < 0.001), and CS (β = 52.53, *SE* = 1.73, *p* < 0.001).

**Table 3.** Path model estimates for the relationship among age, RD, and naming

Naming pictures of everyday objects				
	SLF	ILF	FAT	CS
Age–RD	0.37 (0.009)*	0.08 (0.009)*	0.42 (0.009)*	0.28 (0.01)*
Age–IES	66.96 (1.70)*	52.42 (1.52)*	60.09 (1.70)*	52.53 (1.73)*
RD–IES	−14.09 (1.62)*	2.84 (1.54)	−18.37 (1.66)*	−3.37 (1.67)
RD mediation of Age–IES	−5.16 (0.61)*	0.22 (0.12)	−7.68 (0.71)*	−0.93 (0.46)
Naming abstract control items				
	SLF	ILF	FAT	CS
RD–IES	9.39 (1.78)*	−4.97 (1.77)*	−13.88 (1.96)*	2.04 (1.87)
RD mediation of Age–IES	3.43 (0.67)*	−0.50 (0.20)	−5.83 (0.81)*	0.57 (0.52)

*Note*. Values provided are beta estimates with standard errors in parentheses. *p < 0.006, for statistically significant relationships, the significance threshold accounts for multiple comparisons.

RD from the SLF (β = −14.09, *SE* = 1.62, *p* < 0.001) and FAT (β = −18.37, *SE* = 1.66, *p* < 0.001) were negatively related to picture naming latency. RD of both the SLF (β = −5.16, *SE* = 0.61, *p* < 0.001) and FAT (β = −7.68, *SE* = 0.71, *p* < 0.001) also mediated the relationship between age and picture naming latency. Moreover, for the SLF and FAT, the mediating relationship between naming latencies and RD was significantly stronger than the corresponding path in the CS model (*z* = 5.568, *p* < 0.001; *z* = 7.961, *p* < 0.001, respectively). RD from the ILF and CS were not significantly related to naming latency and did not mediate the age-naming latency relationship (*p*s > 0.01).

Tests of the role of RD on naming control trials revealed that RD from the SLF, ILF, and FAT were related to naming latency (β = 9.39, *SE* = 1.78, *p* < 0.001; β = −4.97, *SE* = 1.77 *p* = 0.005; β = −13.88, *SE* = 1.96, *p* < 0.001, respectively) and that SLF and FAT RD mediated the relationship between age and naming control trials (β = 3.43, *SE* = 0.67, *p* < 0.001; β = −5.83, *SE* = 0.81, *p* < 0.001, respectively), though ILF RD did not (*p* = 0.01). Again, the mediating paths between SLF and FAT RD and naming control trials were significantly stronger than the corresponding path in the CS model (*z* = 3.527, *p* < 0.001, *z* = 6.849, *p* < 0.001, respectively). The CS RD was not significantly related to naming control trials, nor did it significantly mediate the relationship between age and naming (*p*s > 0.1).

## DISCUSSION

A wealth of behavioral research has shown that as we age, word retrieval difficulties frustratingly increase; however, the neural bases of these behaviors are less well understood. One potential neural factor underlying age-related differences in language production is white matter. Here, we tested how white matter relates to age-related language production differences in picture naming by examining DTI-derived metrics that are sensitive to white matter structure. Based on the prior literature, we hypothesized that behaviorally, older adults would have slower and less accurate naming ability. Measuring picture naming with a combined estimate of speed and accuracy (IES), we found that, across all the path models, age was positively correlated with IES values (i.e., increased age was associated with slower accuracy-adjusted naming times). With respect to neural measures and in line with prior literature, we hypothesized that older adults would have lower white matter integrity. Consistent with our hypothesis, we found that age was negatively correlated with FA and positively correlated with RD in the left hemisphere language tracts that we measured (SLF, ILF, and FAT), reflecting age-related white matter deficits in the spatial coherence of estimated fibers (FA) and diffusion across the fibers (RD).

Most importantly, we hypothesized that left-hemisphere dorsal language tracts (SLF, FAT) would have a stronger role in picture naming compared to our control naming condition, and that these white matter tracts would mediate age-related differences in picture naming. Consistent with this, our analysis showed that FA and RD from dorsal language tracts (SLF and FAT) mediated age-related language production deficits in picture naming. Moreover, these significant mediation effects were stronger than the corresponding relations within the CS control tract, which were nonsignificant. The significant mediation effects suggest that white matter connections within dorsal portions of the language network contribute to age-related differences in language production over and above chronological age.

Importantly, FA within the FAT and RD in the SLF, ILF, and FAT also mediated the relationship between chronological age and naming latency of a control condition that placed less demand on word retrieval systems (i.e., saying “picture” to an abstract image). Overall, these findings are consistent with the prior literature (Houston et al., 2019; Madhavan et al., 2014; Stamatakis et al., 2011; Troutman & Diaz, 2019) suggesting that dorsal white matter tracts play a particularly important role in age-related language production deficits. Moreover, the differentiation between picture naming and control trial naming suggests that the SLF has a greater role in higher-order aspects of naming, such as semantic and lexical selection. This is consistent with previous work that has linked posterior regions of the SLF to word retrieval failures (Stamatakis et al., 2011).

Interestingly, the FAT’s sensitivity to both picture naming and control trial naming highlights its importance for speech in general, but it is perhaps more closely related to sensorimotor aspects of speech motor planning or fluency. This is consistent with Catani et al.’s (2013) clinical work that showed greater FAT atrophy among individuals with the nonfluent variety of primary progressive aphasia compared to individuals with the semantic variant of primary progressive aphasia, which is more closely linked to semantic deficits as opposed to naming difficulty per se. Moreover, verbal fluency ability across their entire sample (including healthy controls) was significantly correlated with fiber structure of the FAT. Similarly, Dick et al. (2019) have suggested that the left FAT may support speech motor control and may be involved in motor plan selection during speech. Likewise, in their study of picture-word interference in healthy adults, Troutman and Diaz (2019) found that higher FA and lower RD from dorsal tracts, including the FAT, were linked to better naming performance in younger and older adults. Results from the present study build upon previous literature by providing a direct test of the role of dorsal white matter as a mechanism of age-related deficits in speech production and suggest that the left SLF may be more directly related to lexical or semantic selection, while the left FAT has a role in fluency and speech motor planning.

Critically, the observed mediation effects were specific to language-relevant tracts, and white matter from the CS, as measured by either FA or RD, did not mediate the relationship between age and picture naming behavior. Post hoc comparisons confirmed that the significant mediating paths (e.g., between picture naming and SLF and FAT FA and RD) were significantly different from the nonsignificant effects in the control CS path. Thus, age-related deficits in language white matter tracts have specific cognitive consequences for language production. Some prior studies of cognitive aging have suggested that age-related changes in the properties of white matter have relatively global relations to cognition, particularly executive function and processing speed (Gazes et al., 2016; Hedden et al., 2016; Johnson et al., 2015; Madden et al., 2017, 2020; Salami et al., 2012). Although our results do not speak to cognitive effects more broadly, we were able to differentiate white matter contributions to naming pictures vs. abstract items, and also to differentiate our results within left-hemisphere language tracts vs. a CS control tract. If these effects were due to general effects of cognition, we might have expected less differentiation across conditions and regions in our results.

Related to the discussion of global vs. local effects, we also observed greater specificity in mediating effects of picture naming in our FA measures, as compared to the RD measures. This suggests that RD may be more closely related to sensorimotor aspects of speech production, as opposed to picture naming specifically.

Of course, this study only focused on one aspect of language production (naming), and it is possible that the age-related differences in white matter tracts that we observed may also influence other cognitive abilities. For example, Rizio and Diaz (2016) found that higher FA along the FAT and SLF was linked to better working memory in both older and younger adults, and working memory has been linked both to language comprehension (Caplan & Waters, 2005) and language production (MacDonald, 2013). Future work should test whether white matter from the FAT and SLF contributes independently to age-related declines in both working memory and language production, or whether declines in one of these domains precipitates declines in the other. Moreover, we focused on a subset of language-relevant tracts, and did not include the uncinate fasciculus, which connects orbital frontal regions with anterior temporal regions. This tract may also have a role in picture naming, particularly in the naming of proper nouns (e.g., Papagno, 2011; Papagno et al., 2016), and future work should evaluate its role in production more thoroughly. Additionally, the present results were obtained from educated, high-functioning, community dwelling adults. In many ways, the age-related decline observed here may be smaller than what might be expected in a more socioeconomically diverse sample, as increased education and higher socioeconomic status have been found to have protective effects on neural decline and cognition (e.g., Chan et al., 2018; Farah, 2017; Hurst et al., 2013; Stern et al., 2019).

One unexpected result of this study was the direction of some of the relationships between white matter metrics and naming behaviors. Lower FA and higher RD are typically interpreted as markers of poorer white matter integrity and consequently associated with poorer behavioral outcomes. However, in our mediation models higher SLF FA and lower SLF and FAT RD predicted higher IES values (i.e., slower naming). Bivariate correlations largely confirmed the expected direction of results (i.e., increases in RD = slower naming, increases in FA = faster naming), and path model fit metrics confirmed good model fits for the path models. One consideration is that our dependent variable, efficiency scores, incorporates both response time and accuracy. Overall, the behavioral effects of age were strongest in our accuracy measure and it could be that the efficiency scores were more sensitive to this aspect of the age-related behavioral differences.

A final important consideration when interpreting these and all DTI results is the indirect nature of the white matter measures. Previous studies have confirmed that FA and RD are highly correlated with white matter structure and that they are also influenced by other microstructural properties not considered here (Jones et al., 2013; Wheeler-Kingshott & Cercignani, 2009).

Despite these limitations, these results highlight the importance of white matter in supporting language production, and white matter as one mechanism underlying the commonly observed age-related differences in language production. Consistent with prior reports, we found that increases in age were associated with slower and less accurate picture naming. We also observed well-established, age-related differences in FA and RD measures of white matter integrity. Importantly, these white matter measures from the SLF and FAT mediated the relationship between age and behavioral performance and suggest that left SLF may be important for lexical and semantic selection, while left FAT may have a role for speech fluency or speech motor planning. Data from this broad sample of adults suggest that age-related deficits in dorsal white matter contribute to older adults’ deficits in language production above and beyond effects of age.

## ACKNOWLEDGMENTS

We thank the staff and scientists at the Social, Life, & Engineering Sciences Imaging Center and the Center for Language Science, where the data were collected. This publication was supported by funding from the National Institute on Aging NIH NIA R01 AG034138 (Michele T. Diaz). Sara B. W. Troutman was supported by funding from a National Institute on Aging T32 fellowship while she worked on these analyses (NIH NIA T32 AG049676 to David Almeida and Lynn Martire).

## FUNDING INFORMATION

Michele T. Diaz, National Institute on Aging (https://dx.doi.org/10.13039/100000049), Award ID: R01 AG034138. Sara B. W. Troutman, National Institute on Aging (https://dx.doi.org/10.13039/100000049), Award ID: T32 AG049676.

## AUTHOR CONTRIBUTIONS

**Sara B. W. Troutman**: Formal analysis; Investigation; Visualization; Writing – original draft. **David J. Madden**: Funding acquisition; Writing – review & editing. **Michele T. Diaz**: Conceptualization; Formal analysis; Funding acquisition; Project administration; Supervision; Writing – original draft.

## TECHNICAL TERMS

Magnetic resonance imaging (MRI): A technique that uses strong magnetic fields and radio frequencies to provide images of the brain and other parts of the body.
Diffusion tensor imaging (DTI): An MRI sequence that provides information regarding the rate and directionality of water diffusion. This is often used as an assessment of white matter integrity.
Fractional anisotropy (FA): Reflects the degree to which water diffusion is directional (anisotropic) rather than random or equal in all directions (isotropic). This is most often used as an indicator of fiber tract integrity.
Radial diffusivity (RD): Reflects the rate of diffusivity perpendicular to the principal direction. This is often used as an indicator of myelination.
