# Supplementary material for: Cerebral White Matter Mediation of Age-Related Differences in Picture Naming Across Adulthood
Source: Neurobiol Lang (Camb). 2022 Mar 30;3(2):272–86. doi: 10.1162/nol_a_00065 (PMC9169883; doi:10.1162/nol_a_00065)
Supplement: Supplementary file 1 [file nol-3-2-272-s001.docx]

**Supplemental Materials**

Tractography Procedure

Appropriate SLF tracts were found in 82 participants; Appropriate ILF tracts were found in 80 participants; Appropriate FAT tracts were found in 77 participants; Appropriate CS tracts were found in 74 participants.

**Supplemental Table 1. Tractography Procedures for Representative Tracts**

|  | **Inferior Longitudinal Fasciculus (ILF)** | **Frontal Aslant**  **Tract (FAT)** | **Superior Longitudinal Fasciculus (SLF) III** | **Corticospinal (CS) Tract** |
| --- | --- | --- | --- | --- |
| **Seed** | Temporal Pole | Supplementary Motor Area (SMA) and pre-SMA | Inferior Frontal Gyrus pars opercularis | Cerebral Peduncle |
| **Target** | Inferior Temporal Gyrus, temporo-occipital portion | Inferior Frontal Gyrus, pars opercularis and pars triangularis | Posterior Superior Temporal Gyrus | Precentral Gyrus |
| **Cleaning** | All voxels included in the tracts were ventral to Z = 40 in MNI space to ensure that no tracts traveled too dorsally. | All voxels included in the tracts were dorsal to Z = 40 in MNI space to ensure that no tracts traveled too ventrally. | All voxels included in the tracts were dorsal to Z = 40 in MNI space to ensure that no tracts traveled too ventrally. | All voxels included in the tracts were posterior to Y = 45 in MNI space to ensure that no tracts traveled too anteriorly. |

*All tracts were defined using the Harvard Oxford Cortical Atlas, thresholded at 25%, except for the Cerebral Peduncle, which was defined using the Johns Hopkins University Atlas.

Supplemental Table 2. Model Fit Indices

| Model | CLI | TLI | AIC | BIC |
| --- | --- | --- | --- | --- |
| FA | | | | |
| SLF | 1.00 | 1.00 | 216,918.91 | 216,956.55 |
| ILF | 1.00 | 1.00 | 210,388.48 | 210,426.01 |
| FAT | 1.00 | 1.00 | 203,027.60 | 203,064.92 |
| CS | 1.00 | 1.00 | 195,092.99 | 195,130.09 |
| RD | | | | |
| SLF | 1.00 | 1.00 | 209,690.63 | 209,728.14 |
| ILF | 1.00 | 1.00 | 208,767.39 | 208,804.84 |
| FAT | 1.00 | 1.00 | 200,052.14 | 200,089.44 |
| CS | 1.00 | 1.00 | 193,293.23 | 193,330.32 |

Model fit indices for models of the effect of Fractional Anisotropy (FA) and Radial Diffusivity (RD) from the Superior Longitudinal Fasciculus (SLF), Inferior Longitudinal Fasciculus (ILF), and the Frontal Aslant Tract (FAT). Fit indices include Comparative Fit Index (CLI), Tucker-Lewis Index (TLI), Akaike (AIC) and Bayesian information criterion (BIC). Though, Chi-squared tests were significant for all models (*p* < 0.001), indicating poor model fit, the Chi-squared test is known to be influenced by large sample sizes. Thus, we take the other model fit measures as evidence of adequate model fit.

**Praat Analysis Details**

The Praat script automatically finds words onsets and marks them in a textgrid. Onsets are found using a combination of pitch and intensity cues.

Any stretch of pitch over 100 ms and below 250 Hz, with a preceding stretch of 200 ms below an average of 350 Hz, is flagged as a word candidate. The intensity of this region is then tested. If the average intensity of a 150 ms stretch, beginning from the onset of periodicity, is above 40 dB, this region gets another check. From there, the script searches for the onset of the word by working backward from the proposed onset of periodicity to find where the intensity falls back to the level of the noise in the overall recording (around 30 dB). It searches a region 515 ms long, and once it finds a place where the energy is at the level of the noise, it checks the average intensity of the recording 100 ms downstream of that point to make sure the proposed onset is not a local minimum within a longer onset region.

Once the script has finished marking words, it prompts the user to cycle through each of the proposed word onsets. The user will both check that the onset is placed appropriately and label the word. The word onset markings, corrections, and word labels are automatically saved to a textgrid sharing the name of its parent sound file.
