# Supplementary material for: Cerebral White Matter Mediation of Age-Related Differences in Picture Naming Across Adulthood
Source: Neurobiol Lang (Camb). 2022 Mar 30;3(2):272–86. doi: 10.1162/nol_a_00065 (PMC9169883; doi:10.1162/nol_a_00065)
Supplement: Supplementary file 2 [file nol-3-2-272-s002.docx]

**Supplementary Materials**

Tractography Procedure

Data were preprocessed and analyzed using a combination of MRTRIX and FSL tools (Jenkinson et al., 2012; Smith et al., 2004; Tournier et al., 2019). First, data were denoised using the MRTRIX command dwidenoise. Second, to correct for artifacts such as eddy currents and susceptibility artifacts, data were preprocessed using the MRTRIX command dswipreproc including the FSL Topup option. Next, data were skull-stripped using Brain Extraction Tool (Smith, 2002). Finally, to correct for encoding bias, data were field corrected using the MRTRIX command dwibiascorrect. Voxel-wise tensors and tensor metrics were estimated using the MRTRIX commandsdwi2tensor and tensor2metric, respectively. Data were linearly registered to the MNI152 2mm template using FSL FLIRT (Jenkinson et al., 2002).Seeds and targets were warped into native space using FSL (Greve & Fischl, 2009). Seeds and target are detailed in table 2 and tracts were estimated using the MRTRIX command tckgen using the Tensor_prob algorithm. At each voxel, the Tensor_prob algorithm bootstraps DWI data via trilinear interpolation and the resultant principal eigenvector is used as to guide the streamline at that step. One million iterations were performed for each bootstrap. All of the tckgen default parameters were used. This meant that the tracking procedure terminated when 5000 valid streamlines were selected. A valid selected streamline is one which after beginning at a randomly selected voxel from the seed region continued until reaching a voxel in the target region without exiting the brain or crossing into the opposite hemisphere.

To prevent bias from beginning or ending the tracking procedure in a given seed or target, the tracking algorithm was run twice: once beginning in the seed and ending in the target and once beginning in the target and ending in the seed. This resulted in two tracts for each hemisphere. These tracts were combined using FSLmaths and only voxels common to both tracking directions were considered part of the true tract. Tracts were manually inspected for quality such that each viable tract passed through at least three contiguous slices in the most optimal plane of view for the given tract. Because probabilistic tractography can result in a minority of tracts passing through voxels which are known to not be a part of a given tract based on a priori anatomical knowledge (Catani & de Schotten, 2012), tracts were cleaned in a final step to remove extraneously included voxels based on the criteria presented in the table 2. Representative examples of each tract are displayed in figure 1. Appropriate SLF tracts were found in 82 participants; Appropriate ILF tracts were found in 80 participants; Appropriate FAT tracts were found in 77 participants; Appropriate CS tracts were found in 74 participants. Finally, we used FSLstats to query for average Fractional Anisotropy (FA) and Radial Diffusivity (RD) from each participants’ average SLF, FAT, ILF, and CS.

Supplemental Table 1. Tractography Procedures for Representative Tracts

|  | **Inferior Longitudinal Fasciculus (ILF)** | **Frontal Aslant**  **Tract (FAT)** | **Superior Longitudinal Fasciculus (SLF) III** | **Corticospinal (CS) Tract** |
| --- | --- | --- | --- | --- |
| **Seed** | Temporal Pole | Supplementary Motor Area (SMA) and pre-SMA | Inferior Frontal Gyrus pars opercularis | Cerebral Peduncle |
| **Target** | Inferior Temporal Gyrus, temporo-occipital portion | Inferior Frontal Gyrus, pars opercularis and pars triangularis | Superior Temporal Gyrus | Precentral Gyrus |
| **Cleaning** | All voxels included in the tracts were ventral to Z = 40 in MNI space to ensure that no tracts traveled too dorsally. | All voxels included in the tracts were dorsal to Z = 40 in MNI space to ensure that no tracts traveled too ventrally. | All voxels included in the tracts were dorsal to Z = 40 in MNI space to ensure that no tracts traveled too ventrally. | All voxels included in the tracts were posterior to Y = 45 in MNI space to ensure that no tracts traveled too anteriorly. |

*All tracts were defined using the Harvard Oxford Cortical Atlas, thresholded at 25%, except for the Cerebral Peduncle, which was defined using the Johns Hopkins University Atlas.

Supplemental Table 2. Model Fit Indices

| Model | CLI | TLI | AIC | BIC |
| --- | --- | --- | --- | --- |
| FA | |  |  |  |
| SLF | 1.00 | 1.00 | 216,918.91 | 216,956.55 |
| ILF | 1.00 | 1.00 | 210,388.48 | 210,426.01 |
| FAT | 1.00 | 1.00 | 203,027.60 | 203,064.92 |
| CS | 1.00 | 1.00 | 195,092.99 | 195,130.09 |
| RD |  |  |  |  |
| SLF | 1.00 | 1.00 | 209,690.63 | 209,728.14 |
| ILF | 1.00 | 1.00 | 208,767.39 | 208,804.84 |
| FAT | 1.00 | 1.00 | 200,052.14 | 200,089.44 |
| CS | 1.00 | 1.00 | 193,293.23 | 193,330.32 |

Model fit indices for models of the effect of Fractional Anisotropy (FA) and Radial Diffusivity (RD) from the Superior Longitudinal Fasciculus (SLF), Inferior Longitudinal Fasciculus (ILF), and the Frontal Aslant Tract (FAT). Fit indices include Comparative Fit Index (CLI), Tucker-Lewis Index (TLI), Akaike (AIC) and Bayesian information criterion (BIC). Though, Chi-squared tests were significant for all models (*p* < 0.001), indicating poor model fit, the Chi-squared test is known to be influenced by large sample sizes. Thus, we take the other model fit measures as evidence of adequate model fit.
